# Dietary calcium is inversely associated with hepatitis B virus infection: an analysis of US National Health and Nutrition Examination Survey (NHANES) 2007–2020

**DOI:** 10.1186/s41043-024-00532-4

**Published:** 2024-03-06

**Authors:** Min Zhang, Yuxiao Zhang, Shanjiamei Jiang, Heng Hu, Xinzhi Wang, Fan Yu, Yue’e Huang, Yali Liang

**Affiliations:** 1 School of Public Health, Wannan Medical College, Wuhu, Anhui China; 2 Department of Otolaryngology Head and Neck Surgery, The First Affiliated Hospital of Wannan Medical College, Wuhu, Anhui China

**Keywords:** Dietary calcium intake, Serum calcium, HBV, Infection, NHANES

## Abstract

**Background:**

There have been studies on the relationship between hepatitis B virus (HBV) infection and diet. We hypothesized HBV infection is related to dietary calcium intake, but the evidence is limited. This study aimed to examine whether dietary calcium intake is independently related to HBV infection in the United States population.

**Methods:**

A total of 20,488 participants aged over 20 years from the National Health and Nutrition Examination Survey (NHANES), conducted from 2007 to 2020, were included in this study. Pearson correlation was used to test the association between dietary calcium and serum calcium. The relationships of HBV infection with dietary calcium and serum calcium were assessed by logistic regression models.

**Results:**

There was a weak correlation between dietary calcium and serum calcium (r = 0.048). Logistic regression models indicated that HBV infection had a linear negative correlation with dietary calcium (OR 0.37; 95%CI 0.19, 0.76). For each additional 10 mg dietary calcium, the possibility of HBV infection was reduced by 63%. Hepatitis B positive participants had lower serum calcium content than negative participants. Stratified analysis shown the linear relationship between calcium and HBV infection varied among sex, race/ethnicity, and body mass index.

**Conclusion:**

Our findings demonstrated HBV infection was linearly and inversely correlated with dietary calcium. The current study is expected to offer a fresh perspective on reducing HBV infection.

**Supplementary Information:**

The online version contains supplementary material available at 10.1186/s41043-024-00532-4.

## Background

Hepatitis B virus (HBV) belongs to the liver-tropic DNA virus family, and its infection can induce many liver diseases, such as viral hepatitis, cirrhosis and even liver cancer, which seriously endangers people's physical and mental health [1, 2]. It is reported that there were about 862,000 non-hospitalized patients with chronic hepatitis B virus infection in the United States from 2011 to 2016, and the rate of chronic HBV infection had not dropped significantly since 1999 [3, 4]. According to the estimate of the Centers for Disease Control and Prevention (CDC) [5], 21,600 cases of acute HBV infection occurred in 2018, which posed a significant economic burden on the United States [6]. Although the vaccination of HBV vaccine can effectively prevent its transmission and infection, HBV infection remains one of the key public health issues worldwide [7], including the United States [4]. Therefore, we need to conduct in-depth research to explore more factors affecting HBV infection and provide direction for blocking its transmission and prevalence.

Most studies about the factors affecting HBV infection focus on people's living habits, environment and so on [8, 9]. In recent years, some studies have reported the relationship between dietary nutrients and HBV infection or liver cancer, such as the protective effect of dietary manganese on liver and the harm of fatty acids to liver [10]. However, there is a limited amount of literature on the association of dietary calcium intake with HBV infection. Calcium is one of the important components of the human body and also an important part of bone. It participates in various biological processes of the body, including proliferation, apoptosis, cell senescence and cell signal transduction [11, 12]. Numerous studies have shown that lower calcium intake may affect the metabolic syndrome (diabetes, obesity, hypertension) [13–15], which is closely related to chronic liver disease [16, 17]. Calcium is also closely related to the replication of viruses and can resist viral invasion by eliminating viruses that invade cells [18]. So, calcium may directly or indirectly alter various physiological processes in the body that affect hepatitis B virus infection. We hypothesized that HBV infection is inversely related to dietary calcium intake. This study used the existing National Health and Nutrition Examination Survey (NHANES) data to explore the association between HBV infection and dietary calcium intake, and to analyze the variability about serum calcium levels, so as to provide reference basis for the prevention, control and prognosis of HBV infection.

## Materials and methods

### Data source

NHANES is a survey of population health and nutritional status conducted by the Centers for Disease Control and Prevention in the United States. They used a complex, multistage probability design to conduct a sample survey of the unincorporated civilian population residing in the 50 states and D.C. Comprehensive data collection including demographic, socioeconomic, examination related to health and dietary information have been conducted biennially.

In this study, we selected six consecutive NHANES cycles from 2007 to 2020 due to the lack of Vitamin D (VD) measurements in the data prior to 2007. Besides, data collection was incomplete in the cycle of 2019–2020 owing to the coronavirus disease 2019 (COVID-19) pandemic. Therefore, data collected from 2019 to March 2020 were combined with data from the NHANES 2017–2018 cycle to form a nationally representative sample of NHANES 2017-March 2020 pre-pandemic data. All databases were available from the NHANES website (https://www.cdc.gov/nchs/nhanes/index.htm).

**Fig. 1:**
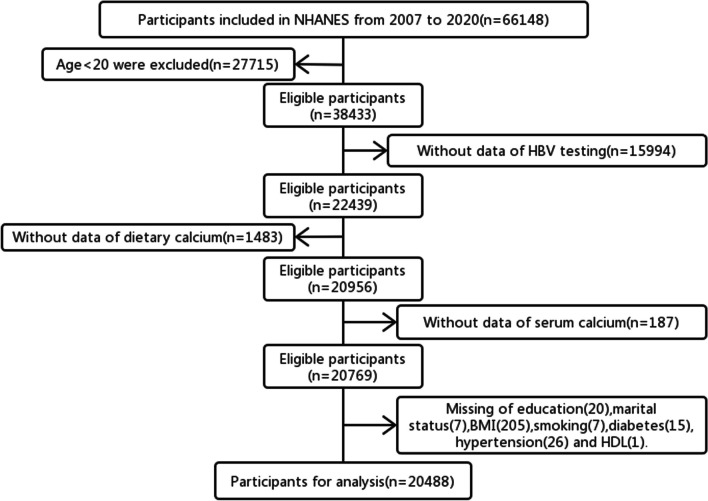
Flow chart for inclusion and exclusion of research subjects

In total, 20,488 participants were enrolled in this study (Fig. 1). The exclusion criteria include: (1) Age under 20 years; (2) Individuals without data of HBV testing; (3) Participants without data of dietary calcium intake; (4) Participants without data of serum calcium; (5) Missing of other variables including education, marital status, BMI, smoking, diabetes, hypertension and HDL.

### HBV infection

The participants' serum samples were collected, processed and frozen (− 30 °C), and then transported to the CDC. The test was carried out by professionals through the VITROS HBsAg reagent kit and VITROS immune diagnostic products HBsAg calibrator on the VITROS ECi/ECiQ immune diagnostic systems and the VITROS 3600 immune diagnostic system. Hepatitis B surface antigen positive participants were considered as HBV carriers or infected. The variable of HBV infection was recorded as a binary variable (1 = infected with HBV; 0 = uninfected with HBV).

### Calcium

Two types of calcium exposure were estimated: dietary calcium intake (mg) and serum calcium (mmol/L). The assessment of dietary calcium was obtained from the 24-h dietary recall questionnaire which was self-reported by the participants. Then the USDA’s Food Surveys Research Group (FSRG) encoded the data and calculated dietary calcium content according to their Food and Nutrient Database for Dietary Studies [19]. Blood samples were collected and stored in mobile devices by certified laboratory professionals. Serum calcium concentrations was determined by Beckman Synchron LX20(Beckman Coulter, Brea, CA) [20].

### Covariates

The covariates involved in this secondary analysis were selected based on previous studies on HBV infection [10, 21], including demographic characteristics, lifestyle and disease history, and other nutrients ingested through food.

We included the following demographic information: sex, age (≥ 20 years old, all responses of participants aged 80 years and older were coded as “80”), race/ethnicity (Mexican American, Other Hispanic, Non-Hispanic White, Non-Hispanic Black, Other Race), body mass index (BMI, calculated as weight in kilograms divided by height in meters squared, and then rounded to one decimal place, kg/m^2^), education level (less than high school diploma, high school, more than high school diploma) and marital status (married or living with partner, widowed or divorced or separated, never married). In addition, we also controlled for smoking (whether smoking more than 100 cigarettes in life), diabetes and hypertension as confounding variables. Disease history information was obtained from participants self-reported data, that was whether they were diagnosed as hypertension or diabetes by doctors (yes or no). Some variables were missing too much, such as drinking alcohol. If the missing values were deleted, it will affect the positive rate of HBV infection, so it was not included in the covariate analysis.

The calculation of other dietary substances intake was the same as that of dietary calcium, which was obtained through the 24-h dietary recall questionnaire. Other indicators mainly included fat (g), fatty acid (Saturated fatty acids, Monounsaturated fatty acids, Polyunsaturated fatty acids, g), vitamin [VB6 (mg), VB12 (µg), VC (mg), VD (µg)], folate (µg), caffeine (mg) and high-density lipoprotein (HDL, mmol/L). The detailed methodology for all the examinations were presented on the NHANES website.

### Statistical analysis

All analyses were performed by using the R 4.2.2 and SPSS 26.0 software. Since the selected dietary and serum calcium data showed skewed distribution after the normality test, we conducted log10 function conversion to facilitate subsequent data analysis. If the continuous variables which were presented as mean ± standard deviation met the test of homogeneity of variance, the differences between groups were tested by independent sample T test (two groups) or one-way ANOVA (more than two groups). Otherwise, the non-parametric Kruskal–Wallis (K–W) test was used. The categorical variables were expressed as percentages, and the inter-group comparison was performed by Chi-square test. We grouped dietary calcium and serum calcium by using quartile in order to estimate differences in HVB infection and covariates among different calcium levels. As for dietary calcium, Q1 is ≤ 522 mg/day; Q2 is 523–803 mg/day; Q3 is 804–1174 mg/day; Q4 is ≥ 1175 mg/day.

As for serum calcium, Q1 is ≤ 2.30 mmol/L; Q2 is 2.31–2.35 mmol/L; Q3 is 2.36–2.40 mmol/L; Q4 is ≥ 2.41 mmol/L. Pearson correlations were estimated to test the relationship between dietary calcium and serum calcium.

We used logistic regression to investigate the linear relationship between HBV infection and dietary calcium or serum calcium. The association was assessed by the odds ratio (OR) with a 95% confidence interval (CI). Three models were established in this study: simple model (without any adjustment), minimum adjustment model (adjusting partial covariates including age, sex, race/ethnicity, education level, marital status, BMI, smoking, diabetes and hypertension), and complete adjustment model (adjusting fat, SFA, MUFA, PUFA, VB6, VB12, VC, VD, folate, caffeine and HDL, in addition to the covariates included in minimum adjustment model). It should be noted that the value of serum calcium after logarithmic conversion was too low, and the data of serum calcium was almost normal distribution through calculation, so we used the unconverted data for logistic regression analysis. Meanwhile, in order to determine whether the logistic regression model had a good fit with the relationship between study variables and dependent variables, we performed restrictive cubic spline (RCS) analysis with four degrees of freedom (knots at 10th, 50th, and 90th percentiles) by adjusting the covariates included in complete adjustment model.

A *p* value of less than 0.05 (two-sided) was considered statistically significant.

## Result

### Participants’ characteristics

The baseline information of this study population, including 163 participants with HBV infection and 20,325 participants without HBV infection, were presented in Table 1. The mean age was 49.28 ± 14.97 and 49.57 ± 17.59 years in each group. Participants with HBV infection were mostly male, mainly from other race groups. They had significantly lower SFA and caffeine intake, while HDL was slightly higher than the uninfected (*p* < 0.05).

**Table 1:** Characteristics of American adult participants from the NHANES (N = 20,488)

	HBsAg (+)	HBsAg (−)	*p* value
N	163	20,325	
Age (years)	49.28 ± 14.97	49.57 ± 17.59	0.808
Sex			
Male	98 (60.12%)	9995 (49.18%)	0.005
Female	65 (39.88%)	10,330 (50.82%)	
Race/ethnicity			
Mexican American	5 (3.07%)	3004 (14.78%)	< 0.001
Other hispanic	7 (4.29%)	2070 (10.18%)	
Non-hispanic white	15 (9.20%)	9033 (44.44%)	
Non-hispanic black	47 (28.83%)	4194 (20.63%)	
Other race	89 (54.60%)	2024 (9.96%)	
Education			
Less than high school diploma	44 (26.99%)	5208 (25.62%)	0.624
High school	32 (19.63%)	4638 (22.82%)	
More than high school diploma	87 (53.37%)	10,479 (51.56%)	
Marital status			
Married or living with partner	100 (61.35%)	12,102 (59.54%)	0.663
Widowed or divorced or separated	38 (23.31%)	4549 (22.38%)	
Never married	25 (15.34%)	3674 (18.08%)	
BMI (kg/m^2^)			
Under/normal weight (≤ 24.9)	74 (45.40%)	5950 (29.27%)	< 0.001
Overweight (25–29.9)	57 (34.97%)	6824 (33.57%)	
Obese (≥ 30)	32 (19.63%)	7551 (37.15%)	
Smoking			
Yes	63 (38.65%)	9214 (45.33%)	0.088
No	100 (61.35%)	11,111 (54.67%)	
Diabetes			
Yes	18 (11.04%)	2488 (12.24%)	0.642
No	145 (88.96%)	17,837 (87.76%)	
Hypertension			
Yes	44 (26.99%)	7322 (36.02%)	0.017
No	119 (73.01%)	13,003 (63.98%)	
Fat (g)	71.64 ± 50.05	78.73 ± 46.28	0.052
SFA (g)	21.67 ± 17.99	25.41 ± 16.53	0.004
MUFA (g)	25.87 ± 18.12	28.37 ± 17.69	0.072
PUFA (g)	17.54 ± 12.91	17.93 ± 12.14	0.680
VB6 (mg)	2.13 ± 2.00	2.05 ± 1.60	0.506
VB12 (µg)	4.73 ± 4.93	5.08 ± 6.46	0.493
VC (mg)	89.23 ± 89.23	85.18 ± 98.48	0.601
VD (µg)	4.68 ± 5.93	4.62 ± 5.60	0.885
Folate (µg)	395.44 ± 214.39	397.83 ± 247.68	0.902
Caffeine (mg)	116.74 ± 155.17	154.03 ± 207.61	0.003
HDL (mmol/L)	1.42 ± 0.38	1.36 ± 0.41	0.042
Log10 dietary calcium (mg)	2.77 ± 0.30	2.88 ± 0.28	< 0.001
Log10 serum calcium (mmol/L)	0.37 ± 0.02	0.37 ± 0.02	0.015

*BMI* body mass index, *SFA* saturated fatty acids, *MUFA* monounsaturated fatty acids, *PUFA* polyunsaturated fatty acids, *VB6* vitamin B6, *VB12* vitamin B12, *VC* vitamin C, *VD* vitamin D, *HDL* high-density lipoprotein. Continuous variables were represented by mean ± standard deviation, and inter group differences were tested using independent sample t-test or the non parametric Kruskal Wallis (K–W) test. The categorical variables were expressed as percentages, and the inter-group comparison was performed by Chi-square test

At the same time, we also listed the inter-group differences of covariates by dietary calcium intake and serum calcium grouping (Additional file 1: Tables S1, S2). In the analysis of the dietary calcium, the participants in Q4 group were younger. They had higher intake of fat, saturated fatty acids, monounsaturated fatty acids, polyunsaturated fatty acids, folate, vitamin B6, B12, C and D, while the intake of HDL was slightly lower (*p* < 0.001). Moreover, female participants were more than men in the first three groups, and participants who had a history of diabetes and hypertension were the least in Q4 group (*p* < 0.001). Finally, these participants in the Q4 group showed lower rates of HBV infection than the other groups (*p* < 0.001). The absolute risk difference (ARD) between Q1 group and Q4 group was 0.73%. As for serum calcium, no significant differences were detected on BMI, smoking, the intake of fat, saturated fatty acids, monounsaturated fatty acids, polyunsaturated fatty acids, VB12, VC, folate and Caffeine (*p* > 0.05).

### Correlation between dietary calcium and serum calcium

**Fig. 2:**
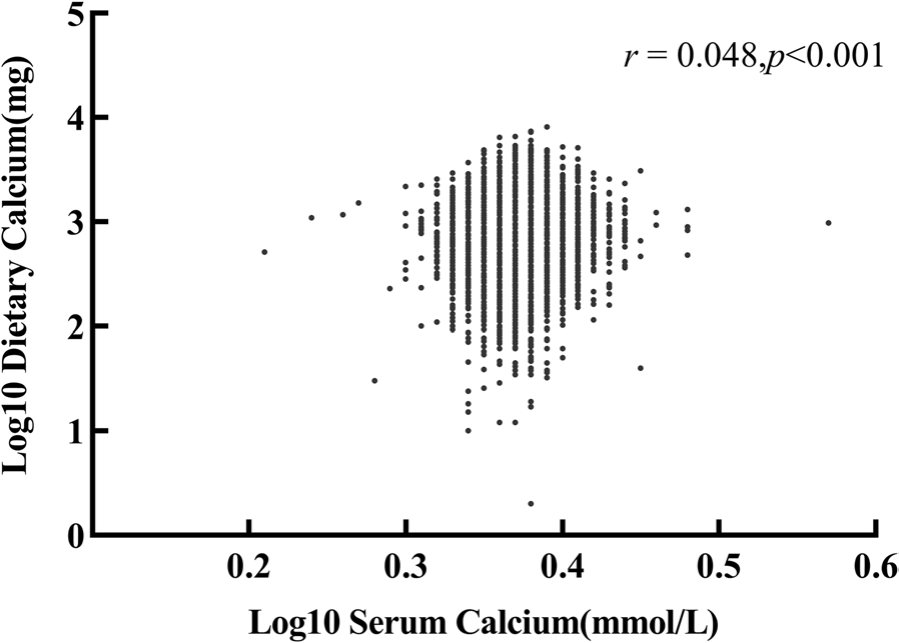
Scatter plot of dietary calcium versus serum calcium (correlation: r = 0.048, *p* value < 0.001)

We used Pearson correlations to assess the relationship between dietary calcium and serum calcium. The correlation coefficient was 0.048 which showed a low correlation (Fig. 2). It indicated that a higher dietary calcium intake does not necessarily represent higher serum calcium.

### Linear relationship between HBV infection and dietary calcium intake, serum calcium

The results of logistic regression about the relationship between HBV infection status and dietary calcium intake and serum calcium were shown in Table 2. It can be seen from the table that the intake of dietary calcium was negatively correlated with HBV infection (OR 0.30; 95%CI 0.19, 0.48) in Model 1. The same trend occurred in Model 2 (OR 0.49; 95%CI 0.29, 0.83) and Model 3 (OR 0.37; 95%CI 0.19, 0.76). The serum calcium content decreased in patients with HBV infection regardless of modulation of covariates.

**Table 2:** Association between HBV infection and calcium (dietary and serum calcium) in different models

	Log10 dietary calcium intake		Serum calcium concentration	
	OR (95%CI)	*p* value	OR (95%CI)	*p* value
Model 1	0.30 (0.19, 0.48)	< 0.001	0.12 (0.02, 0.66)	0.015
Model 2	0.49 (0.29, 0.83)	0.008	0.12 (0.02, 0.69)	0.018
Model 3	0.37 (0.19, 0.76)	0.006	0.11 (0.02, 0.63)	0.013

*OR* odds ratio, *CI* confidence interval. Model 1: No covariates were adjusted. Model 2: Adjusted for age, sex, race/ethnicity, education level, marital status, BMI, smoking, diabetes and hypertension. Model 3: Adjusted for fat, SFA, MUFA, PUFA, VB6, VB12, VC, VD, folate, caffeine and HDL, in addition to the covariates included in Model 2

**Fig. 3:**
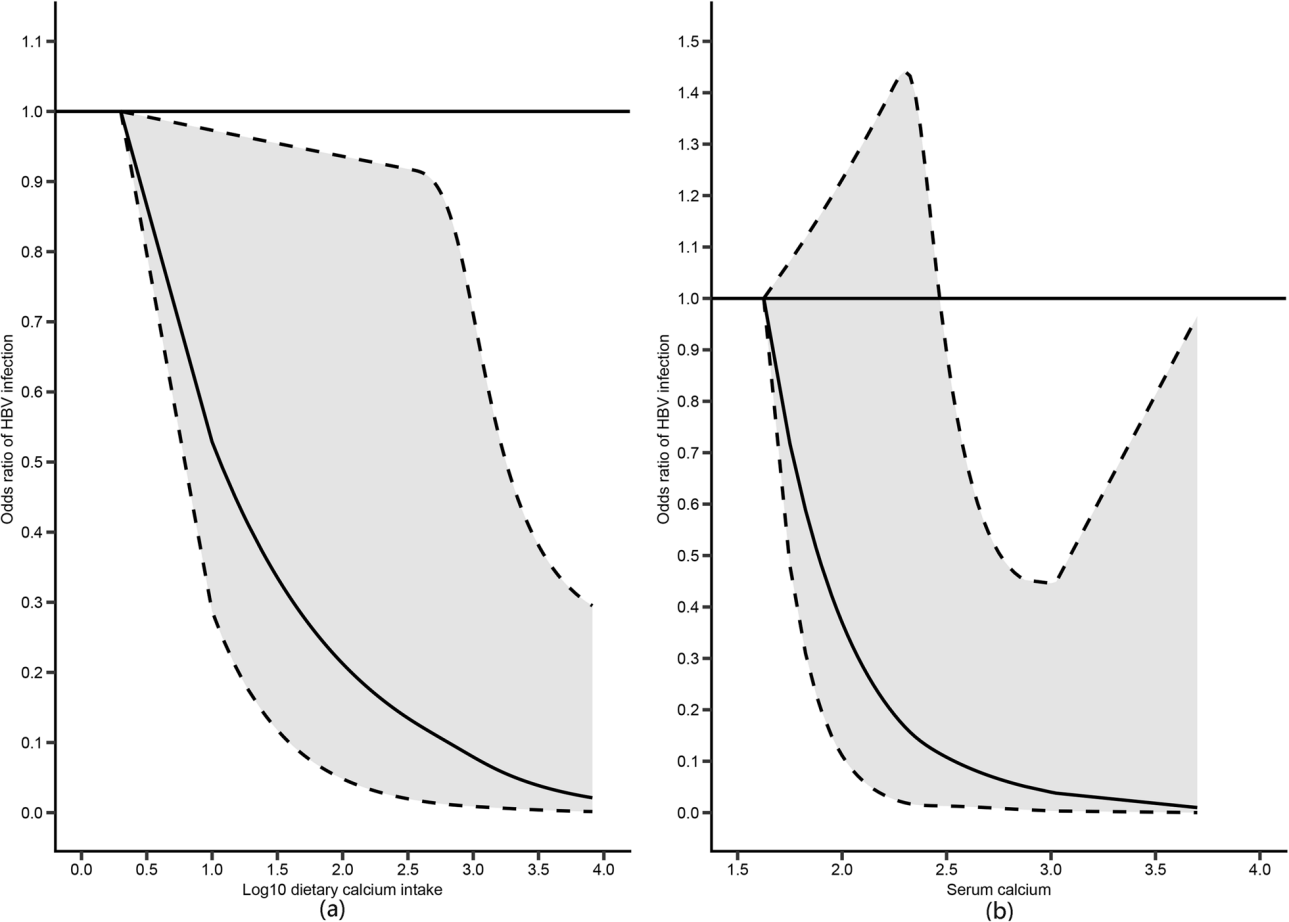
Restricted cubic spline (RCS) analysis of predicted dietary and serum calcium for the risk of HBV infection. We used RCS to further explore linear association and all analysis were adjusted for age, sex, race/ethnicity, education level, marital status, BMI, smoking, diabetes,hypertension, fat, SFA, MUFA, PUFA, VB6, VB12, VC, VD, folate, caffeine and HDL. **a** The association of dietary calcium and HBV infection (*p* for all < 0.016, *p* for non-linearity = 0.642). **b** The association of serum calcium and HBV infection (*p* for all < 0.028, *p* for non-linearity = 0.836). HBV: Hepatitis B Virus

We subsequently used restrictive cubic splines (RCS) to flexibly model and visualize the relation between HBV infection status and predicted dietary calcium, serum calcium (Fig. 3). The results showed that there was a linear relationship between dietary calcium and HBV infection (*p* for all < 0.0162), but no non-linear relationship (*p* for non-linearity = 0.6422). So was serum calcium (*p* for all < 0.0281, *p* for non-linearity = 0.8362).

### Stratified analysis of HBV infection versus dietary calcium and serum calcium

**Fig. 4:**
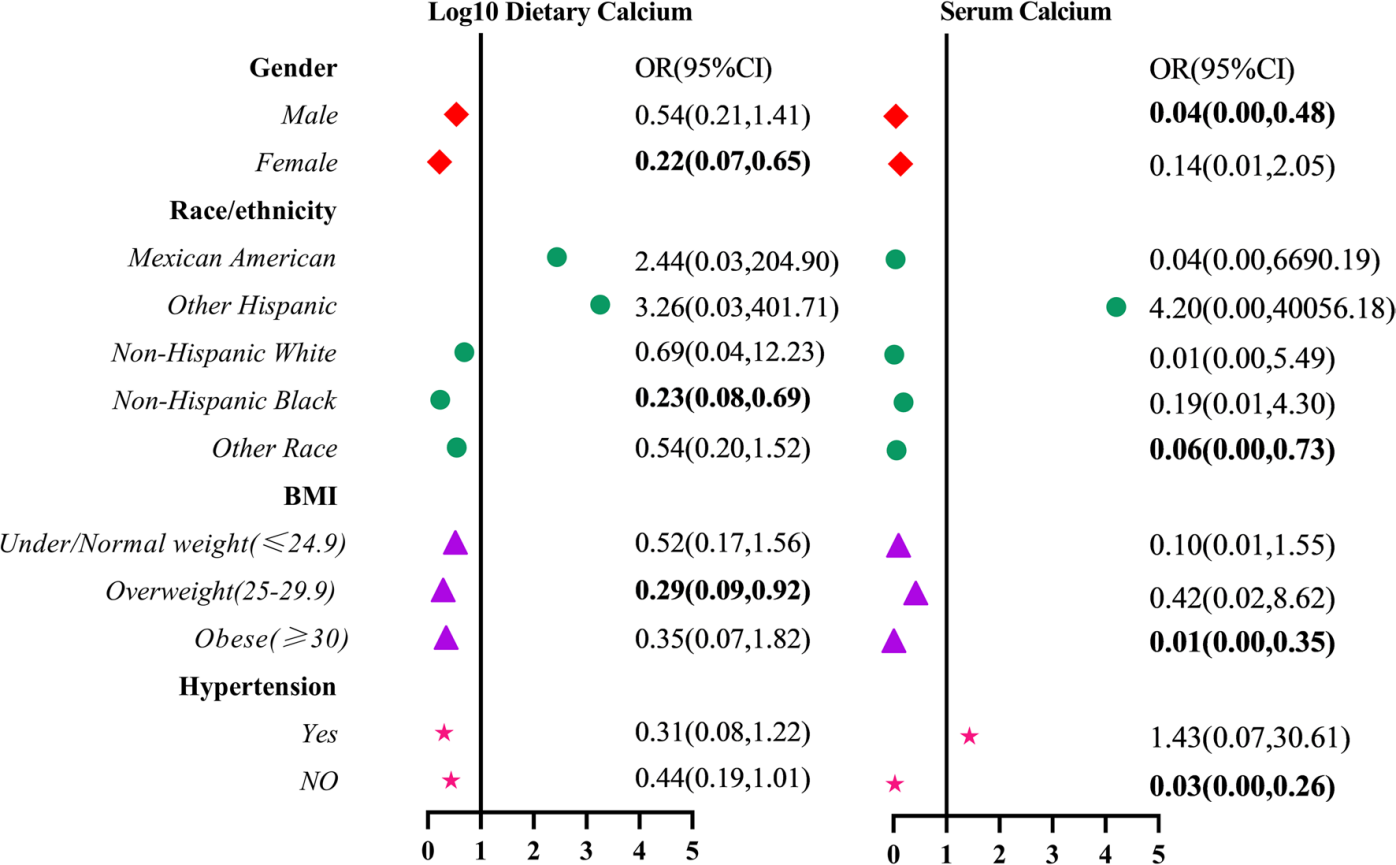
Stratified analysis for the relationship between HBV infection and calcium (dietary and serum calcium)

The factors that may influence the correlation results were stratified based on Model 3 (adjusting age, sex, race/ethnicity, education level, marital status, BMI, smoking, diabetes,hypertension, fat, SFA, MUFA, PUFA, VB6, VB12, VC, VD, folate, caffeine and HDL). The analysis results were shown in Fig. 4. For sex stratification, HBV infection in male have lower serum calcium levels than uninfected participants (OR 0.04; 95%CI 0.00, 0.48); dietary calcium intake was independent of its infection status. HBV infection in female was inversely associated with dietary calcium intake (OR 0.22; 95%CI 0.07, 0.65), and serum calcium was meaningless.

For racial stratification, Only Non-Hispanic Black individuals with HBV infection were associated with dietary calcium intake (OR 0.23; 95%CI 0.08, 0.69). As for BMI stratification, dietary calcium intake only played a role in overweight people (OR 0.29; 95%CI 0.09, 0.92). The serum calcium levels of HBV-infected participants were lower in obese individuals (OR 0.01; 95%CI 0.00, 0.35), and there was no difference in serum calcium levels between normal and overweight individuals.

## Discussion

We used the data of six cycles in the NHANES database to explore the association between HBV infection and dietary calcium. From 2007 to 2020, 0.8% of the participants were positive for hepatitis B surface antigen in the survey. The results have confirmed the hypothesis for that dietary calcium intake was significantly and negatively associated with HBV infection. From the aspect of mechanism, the reason may be that HBV infection is related to oxidative stress in vivo [22]. The occurrence of gene mutations is one of the main mechanisms by which HBV survives and evades host immune responses, and oxidative stress may lead to the occurrence and development of mutations. Elevated antioxidant levels and inadequate antioxidant defense are closely associated with HBV gene mutation [23]. Calcium also plays an important role in oxidative stress reactions. Studies have shown that high-calcium diets can suppress the occurrence of oxidative stress reactions in the human body [24, 25]. Therefore, dietary calcium can affect HBV infection by regulating oxidative stress. Virus replication is also one of the possible reasons for the negative correlation between dietary calcium intake and HBV infection. Studies have shown that calcium is essential for viral replication [26]. In addition, HBx protein can activate cellular signaling pathways during HBV replication, which are necessary for viral infection, and calcium signaling pathway plays an important role in these pathways [27]. Our study also found that the serum calcium content of the hepatitis B positive participants was lower than the hepatitis B negative participants. The portion of calcium that people consume through food is absorbed by the small intestine and enters the bloodstream to become blood calcium. This also further confirmed the hypothesis that dietary calcium intake was inversely associated with HBV infection.

From the results, it can be seen that there was a very weak correlation between dietary calcium and serum calcium. However, Gebreyohannes' study showed no correlation between dietary calcium and serum calcium, while Byrne FN's study showed a strong correlation [28, 29]. Because diet is not the only factor that affects serum levels. The homeostasis of serum calcium metabolism also depends on other factors such as hormones, and so the results may vary slightly from study to study. We also found that individual and dietary factors increased the protective effect of dietary calcium against HBV infection. Because dietary calcium must go through a series of complex pathways in the process of ingestion, including absorption, consumption and storage, it is susceptible to other external factors [30]. It is suggested that more attention should be paid to the intake of dietary calcium in daily diet, such as dairy products, dark green leafy vegetables, nuts, green beans and so on [31]. It can be taken in combination with foods rich in vitamin D [32], and more sun exposure is also one of the ways to promote calcium absorption [19].

Sex, race/ethnicity and body mass index differences were observed in our study. In the study of dietary calcium, we found that dietary calcium intake to the extent reduced the risk of HBV infection in women, but there was no significant difference in men. It may be because some poor lifestyle habits in men have lowered their immune levels, such as frequent dining out, drinking alcohol and so on, which fails to reduce the risk of HBV infection even if dietary calcium is consumed [18]. Moreover, men's higher life pressure will increase oxidative stress and be more susceptible to HBV infection [33]. The serum calcium levels of male HBV infected individuals were lower than those of non infected individuals, while there was no significant difference in serum calcium levels between female HBsAg positive and negative populations. This sex difference in the protective effect of serum calcium can be explained by women's sex hormones and menstrual cycle. It was reported that serum calcium levels change at different stages of the menstrual cycle under the influence of sex hormones [34]. Individual differences may affect the measurement of serum calcium, thereby masking the phenomenon of lower serum calcium levels in female infected individuals. In addition, estrogen reduces serum calcium by enhancing the absorption of intestinal calcium [35], pregnancy and lactation can also lower serum calcium levels in women [36]. The diversity between race/ethnicity may be related to different dietary habits, lifestyles, and economic levels [37]. Studies have shown that overweight and obesity are one of the important factors affecting HBV infection, and high dietary calcium intake can reduce obesity to some extent, thereby reducing the risk of infection [38].

Our study had the following strengths: (1) The data source was reliable and had a large sample size. NHANES is a series of well-designed investigations with a high-quality assurance and quality control process by CDC of the United States, which can ensure the timeliness and high quality of the data [39]. (2) The analysis was comprehensive. We not only investigated the relationship between dietary calcium and HBV infection, but also explored differences in serum calcium levels between HBsAg positive and negative individuals. (3). According to previous studies, this is the first time that the linear relationships between calcium and HBV infection have been found. And no matter in which model they were stable linear association. (4) RCS analysis was used to further verify the existence of linear relationship between HBV infection and dietary calcium. (5) The factors that may affect the results were analyzed hierarchically to further explore the impact of different groupings on the research results.

However, there were some limitations to our findings. First of all, the cross-sectional study could not obtain exact causal relationships and the conclusive evidence was weak. Secondly, dietary calcium data and disease history were self-reported by participants and there may be a memory bias. People's reported height tended to be higher than the actual value, while their weight tended to be lower than the actual value. Thirdly, female participants were at different stages of the menstrual cycle, which affected the serum calcium measurements. For example, an increase in female sex hormones during menstruation may lead to a decrease in serum calcium levels [34]. Finally, serum calcium only reflects the recent status and cannot represent the long-term calcium level, but no more suitable measurement index has been found yet [40]. Although our research had some shortcomings, the results indicated that it is important to pay attention to the appropriate intake of dietary calcium in daily life, and adjust dietary habits in a timely manner to prevent potential problems.

## Conclusions

In conclusion, we can see that HBV infection may be associated with reduced dietary calcium intake. The correlations were sex-different due to the different living habits and hormones in men and women. Race/ethnic differences are also a direction worth studying. More prospective studies are needed to further verify the associations between dietary calcium and HBV infection, to lay a certain foundation for exploring the relationship between other dietary elements such as SFA, caffeine, HDL, and HBV infection, so as to provide a reference for the prevention, control and prognosis of HBV infection.

### Supplementary Information

**Additional file 1: Table S1. **Characteristics of American adult participants from the NHANES among different groups of dietary calcium intake. **Table S2. **Characteristics of American adult participants from the NHANES among different groups of serum calcium.

## Data Availability

The data that support the findings of this study are openly available in NHANES at https://www.cdc.gov/nchs/nhanes/index.htm.

## References

[1] Yan LB, Liao J, Han N, Zhou LY, Wang XE, Wang YJ, Tang H (2020). Association between hepatitis B virus infection and metabolic syndrome in Southwest China: a cross-sectional study. Sci Rep.

[2] Du Y, Zhang S, Hu M, Wang Q, Liu N, Shen H, Zhang Y, Yan D, Zhang M (2019). Association between hepatitis B virus infection and chronic kidney disease: a cross-sectional study from 3 million population aged 20 to 49 years in rural China. Medicine (Baltimore).

[3] Patel EU, Thio CL, Boon D, Thomas DL, Tobian AAR (2019). Prevalence of hepatitis B and hepatitis D virus infections in the United States, 2011–2016. Clin Infect Dis.

[4] Roberts H, Ly KN, Yin S, Hughes E, Teshale E, Jiles R (2021). Prevalence of HBV infection, vaccine-induced immunity, and susceptibility among at-risk populations: US HOUSEHOLDS, 2013–2018. Hepatology.

[5] Roberts H, Jiles R, Harris AM, Gupta N, Teshale E (2021). Incidence and prevalence of sexually transmitted hepatitis B, United States, 2013–2018. Sex Transm Dis.

[6] Park H, Jeong D, Nguyen P, Henry L, Hoang J, Kim Y, Sheen E, Nguyen MH (2018). Economic and clinical burden of viral hepatitis in California: a population-based study with longitudinal analysis. PLoS ONE.

[7] Schmit N, Nayagam S, Thursz MR, Hallett TB (2021). The global burden of chronic hepatitis B virus infection: comparison of country-level prevalence estimates from four research groups. Int J Epidemiol.

[8] Hu H, Shen Y, Hu M, Zheng Y, Xu K, Li L (2021). Incidence and influencing factors of new hepatitis B infections and spontaneous clearance: a large-scale, community-based study in China. Front Med (Lausanne).

[9] Le TV, Vu TTM, Dang AK, Vu GT, Nguyen LH, Nguyen BC, Tran TH, Tran BX, Latkin CA, Ho CSH (2019). Understanding risk behaviors of Vietnamese adults with chronic hepatitis B in an urban setting. Int J Environ Res Public Health.

[10] Yang WS, Zeng XF, Liu ZN, Zhao QH, Tan YT, Gao J, Li HL, Xiang YB (2020). Diet and liver cancer risk: a narrative review of epidemiological evidence. Br J Nutr.

[11] Smaili S, Hirata H, Ureshino R, Monteforte PT, Morales AP, Muler ML, Terashima J, Oseki K, Rosenstock TR, Lopes GS (2009). Calcium and cell death signaling in neurodegeneration and aging. An Acad Bras Cienc.

[12] Cormick G, Belizán JM (2019). Calcium intake and health. Nutrients.

[13] Woo HW, Lim YH, Kim MK, Shin J, Lee YH, Shin DH, Shin MH, Choi BY (2020). Prospective associations between total, animal, and vegetable calcium intake and metabolic syndrome in adults aged 40 years and older. Clin Nutr.

[14] Han D, Fang X, Su D, Huang L, He M, Zhao D, Zou Y, Zhang R (2019). Dietary calcium intake and the risk of metabolic syndrome: a systematic review and meta-analysis. Sci Rep.

[15] Park S, Kim K, Lee BK, Ahn J (2021). A healthy diet rich in calcium and vitamin C is inversely associated with metabolic syndrome risk in Korean adults from the KNHANES 2013–2017. Nutrients.

[16] Åberg F, Byrne CD, Pirola CJ, Männistö V, Sookoian S (2023). Alcohol consumption and metabolic syndrome: clinical and epidemiological impact on liver disease. J Hepatol.

[17] Ren H, Wang J, Gao Y, Yang F, Huang W (2019). Metabolic syndrome and liver-related events: a systematic review and meta-analysis. BMC Endocr Disord.

[18] Kumar P, Kumar M, Bedi O, Gupta M, Kumar S, Jaiswal G, Rahi V, Yedke NG, Bijalwan A, Sharma S (2021). Role of vitamins and minerals as immunity boosters in COVID-19. Inflammopharmacology.

[19] Shen X, Gu X, Liu YY, Yang L, Zheng M, Jiang L (2023). Association between dietary calcium and depression among American adults: National health and nutrition examination survey. Front Nutr.

[20] Chen Z, Xu J, Ye P, Xin X (2023). Nonlinearity association of serum calcium with the risk of anaemia in US adults. Hematology.

[21] El-Serag HB (2012). Epidemiology of viral hepatitis and hepatocellular carcinoma. Gastroenterology.

[22] Popa GL, Popa MI (2022). Oxidative stress in chronic hepatitis B-an update. Microorganisms.

[23] Feng JF, Yang YW, Wang D, Tang J, Xie G, Fan LY (2017). Relationship between oxidative stress in patients with HBV-induced liver disease and HBV genotype/drug-resistant mutation. Front Lab Med.

[24] Zemel MB, Sun X (2008). Dietary calcium and dairy products modulate oxidative and inflammatory stress in mice and humans. J Nutr.

[25] Zemel MB (2009). Proposed role of calcium and dairy food components in weight management and metabolic health. Phys Sportsmed.

[26] Rahman SK, Kerviel A, Mohl BP, He Y, Zhou ZH, Roy P (2020). A calcium sensor discovered in bluetongue virus nonstructural protein 2 is critical for virus replication. J Virol.

[27] Bouchard MJ, Puro RJ, Wang L, Schneider RJ (2003). Activation and inhibition of cellular calcium and tyrosine kinase signaling pathways identify targets of the HBx protein involved in hepatitis B virus replication. J Virol.

[28] Gebreyohannes RD, Abdella A, Ayele W, Eke AC (2021). Association of dietary calcium intake, total and ionized serum calcium levels with preeclampsia in Ethiopia. BMC Pregnancy Childbirth.

[29] Byrne FN, Kinsella S, Murnaghan DJ, Kiely M, Eustace JA (2009). Lack of correlation between calcium intake and serum calcium levels in stable haemodialysis subjects. Nephron Clin Pract.

[30] Shkembi B, Huppertz T (2021). Calcium absorption from food products: food matrix effects. Nutrients.

[31] Mäkinen OE, Wanhalinna V, Zannini E, Arendt EK (2016). Foods for special dietary needs: non-dairy plant-based milk substitutes and fermented dairy-type products. Crit Rev Food Sci Nutr.

[32] Herdea A, Ionescu A, Dragomirescu MC, Ulici A (2023). Vitamin D-a risk factor for bone fractures in children: a population-based prospective case-control randomized cross-sectional study. Int J Environ Res Public Health.

[33] Gombart AF, Pierre A, Maggini S (2020). A review of micronutrients and the immune system-working in harmony to reduce the risk of infection. Nutrients.

[34] Dullo P, Vedi N (2008). Changes in serum calcium, magnesium and inorganic phosphorus levels during different phases of the menstrual cycle. J Hum Reprod Sci.

[35] Van Cromphaut SJ, Rummens K, Stockmans I, Van Herck E, Dijcks FA, Ederveen AG, Carmeliet P, Verhaeghe J, Bouillon R, Carmeliet G (2003). Intestinal calcium transporter genes are upregulated by estrogens and the reproductive cycle through vitamin D receptor-independent mechanisms. J Bone Miner Res.

[36] Farhud D, Zarif-Yeganeh M, Mehrabi A, Afshari AR, Rokni MB, Majidi K, Jalali M, Amir Zargar AA, Sarafnejad A, Sadeghipour HR (2022). A retrospective study of serum calcium status in Tehran, Iran (105,128 samples, from 2009-2018). Iran J Public Health.

[37] Malek AM, Newman JC, Hunt KJ, Marriott BP (2019). Race/ethnicity, enrichment/fortification, and dietary supplementation in the U.S. population, NHANES 2009–2012. Nutrients.

[38] Moraes ABV, Veiga GV, Azeredo VB, Sichieri R, Pereira RA (2022). High dietary calcium intake and low adiposity: findings from a longitudinal study in Brazilian adolescents. Cad Saude Publica.

[39] Ahluwalia N, Dwyer J, Terry A, Moshfegh A, Johnson C (2016). Update on NHANES dietary data: focus on collection, release, analytical considerations, and uses to inform public policy. Adv Nutr.

[40] Hua Y, Liu HL, Sun JY, Kong XQ, Sun W, Xiong YQ (2021). Association between serum calcium and the prevalence of hypertension among US adults. Front Cardiovasc Med.

