# Supplementary material for: Dietary calcium is inversely associated with hepatitis B virus infection: an analysis of US National Health and Nutrition Examination Survey (NHANES) 2007–2020
Source: J Health Popul Nutr. 2024 Mar 6;43:38. doi: 10.1186/s41043-024-00532-4 (PMC10916236; doi:10.1186/s41043-024-00532-4)
Supplement: Supplementary file 1 — Additional file 1: Table S1. Characteristics of American adult participants from the NHANES among different groups of dietary calcium intake. Table S2. Characteristics of American adult participants from the NHANES among different groups of serum calcium. [file 41043_2024_532_MOESM1_ESM.docx]

# Supplementary Materials

# Dietary calcium is inversely associated with hepatitis B virus infection: An analysis of US National Health and Nutrition Examination Survey (NHANES) 2007 to 2020

**Supplementary Table 1-** Characteristics of American adult participants from the NHANES among different groups of dietary calcium intake.

|  | **Dietary calcium intake(mg/day)** | | | |  |
| --- | --- | --- | --- | --- | --- |
|  | **Q1(≤522)** | **Q2(523-803)** | **Q3(804-1174)** | **Q4(≥1175)** | **P-value** |
| Age (years) | 51.6±17.54 | 50.98±17.72 | 49.61±17.5 | 46.06±17 | <0.001 |
| Sex |  |  |  |  |  |
| Male | 2176(42.29%) | 2331(45.66%) | 2493(48.70%) | 3093(60.43%) | <0.001 |
| Female | 2970(57.71%) | 2774(54.34%) | 2626(51.30%) | 2025(39.57%) |  |
| Race/ethnicity |  |  |  |  |  |
| Mexican American | 650(12.63%) | 737(14.44%) | 794(15.51%) | 828(16.18%) | <0.001 |
| Other Hispanic | 523(10.16%) | 532(10.42%) | 523(10.22%) | 499(9.75%) |  |
| Non-Hispanic White | 1830(35.56%) | 2178(42.66%) | 2363(46.16%) | 2677(52.31%) |  |
| Non-Hispanic Black | 1478(28.70%) | 1074(21.00%) | 934(18.20%) | 755(14.80%) |  |
| Other Race | 665(12.92%) | 584(11.44%) | 505(9.87%) | 359(7.01%) |  |
| Education |  |  |  |  |  |
| Less than high school diploma | 1650(32.06%) | 1276(25.00%) | 1192(23.29%) | 1134(22.16%) | <0.001 |
| High school | 1251(24.31%) | 1177(23.06%) | 1123(21.94%) | 1119(21.86%) |  |
| More than high school diploma | 2245(43.63%) | 2652(51.95%) | 2804(54.78%) | 2865(55.98%) |  |
| Marital status |  |  |  |  |  |
| Married or living with partner | 2893(56.22%) | 3073(60.20%) | 3080(60.17%) | 3156(61.66%) | <0.001 |
| Widowed or divorced or separated | 1341(26.06%) | 1171(22.94%) | 1147(22.41%) | 928(18.13%) |  |
| Never married | 912(17.72%) | 861(16.87%) | 892(17.43%) | 1034(20.20%) |  |
| BMI (kg/m^2^) |  |  |  |  |  |
| Under/Normal weight (≤24.9) | 1543(29.98%) | 1452(28.44%) | 1549(30.26%) | 1480(28.92%) | 0.237 |
| Overweight (25-29.9) | 1678(32.61%) | 1737(34.03%) | 1729(33.78%) | 1737(33.94%) |  |
| Obese (≥30) | 1925(37.41%) | 1916(37.53%) | 1841(35.96%) | 1901(37.14%) |  |
| Smoking |  |  |  |  |  |
| Yes | 2454(47.69%) | 2307(45.19%) | 2222(43.41%) | 2294(44.82%) | <0.001 |
| No | 2692(52.31%) | 2798(54.81%) | 2897(56.59%) | 2824(55.18%) |  |
| Diabetes |  |  |  |  |  |
| Yes | 754(14.65%) | 667(13.07%) | 610(11.92%) | 475(9.28%) | <0.001 |
| No | 4392(85.35%) | 4438(86.93%) | 4509(88.08%) | 4643(90.72%) |  |
| Hypertension |  |  |  |  |  |
| Yes | 2066(40.15%) | 1910(37.41%) | 1816(35.48%) | 1574(30.75%) | <0.001 |
| No | 3080(59.85%) | 3195(62.59%) | 3303(64.52%) | 3544(69.25%) |  |
| Fat (g) | 50.91±29.26 | 69.42±34.4 | 81.95±37.22 | 112.55±56.06 | <0.001 |
| SFA (g) | 14.63±8.89 | 21.18±10.84 | 26.46±12.21 | 39.28±20.54 | <0.001 |
| MUFA (g) | 18.98±11.95 | 25.36±13.92 | 29.56±15.1 | 39.56±21.54 | <0.001 |
| PUFA (g) | 12.65±8.84 | 16.68±10.42 | 18.63±11.08 | 23.78±14.72 | <0.001 |
| VB6 (mg) | 1.41±1.07 | 1.84±1.3 | 2.17±1.6 | 2.78±1.97 | <0.001 |
| VB12 (µg) | 2.93±6.08 | 4.22±6.04 | 5.32±4.78 | 7.86±7.54 | <0.001 |
| VC (mg) | 56.92±76.49 | 76.28±81.75 | 92.09±98.34 | 115.7±121.18 | <0.001 |
| VD (µg) | 1.97±3.68 | 3.45±4.41 | 4.84±4.74 | 8.24±6.96 | <0.001 |
| Folate (µg) | 246.24±143.69 | 348.13±171.4 | 422.71±203.97 | 574.85±309.69 | <0.001 |
| Caffeine (mg) | 139.83±205.35 | 145.94±177.76 | 154.5±202.45 | 174.73±237.47 | <0.001 |
| HDL (mmol/L) | 1.37±0.43 | 1.37±0.41 | 1.37±0.41 | 1.33±0.40 | <0.001 |
| HBV infection status |  |  |  |  |  |
| Positive | 62(1.20%) | 47(0.92%) | 30(0.59%) | 24(0.47%) | <0.001 |
| Negative | 5084(98.80%) | 5058(99.08%) | 5089(99.41%) | 5094(99.53%) |  |

Dietary calcium intake was grouped by quartile.

Abbreviation: BMI, body mass index; HBV, Hepatitis B Virus; SFA, Saturated fatty acids; MUFA, Monounsaturated fatty acids; PUFA, Polyunsaturated fatty acids; VB6, vitamin B6; VB12, vitamin B12; VC, vitamin C; VD, vitamin D; HDL, high-density lipoprotein.

**Supplementary Table 2-** Characteristics of American adult participants from the NHANES among different groups of serum calcium.

|  | **Serum Calcium(mmol/L)** | | | |  |
| --- | --- | --- | --- | --- | --- |
|  | **Q1(≤2.30)** | **Q2(2.31-2.35)** | **Q3(2.36-2.40)** | **Q4(≥2.41)** | **P-value** |
| Age (years) | 50.08±16.83 | 48.97±17.29 | 49.01±17.98 | 49.95±18.41 | 0.001 |
| Sex |  |  |  |  |  |
| Male | 2890(43.85%) | 2359(49.97%) | 2288(52.80%) | 2556(52.77%) | <0.001 |
| Female | 3700(56.15%) | 2362(50.03%) | 2045(47.20%) | 2288(47.23%) |  |
| Race/ethnicity |  |  |  |  |  |
| Mexican American | 1129(17.13%) | 727(15.40%) | 613(14.15%) | 540(11.15%) | <0.001 |
| Other Hispanic | 697(10.58%) | 506(10.72%) | 424(9.79%) | 450(9.29%) |  |
| Non-Hispanic White | 2742(41.61%) | 2089(44.25%) | 1948(44.96%) | 2269(46.84%) |  |
| Non-Hispanic Black | 1269(19.26%) | 927(19.64%) | 917(21.16%) | 1128(23.29%) |  |
| Other Race | 753(11.43%) | 472(10.00%) | 431(9.95%) | 457(9.43%) |  |
| Education |  |  |  |  |  |
| Less than high school diploma | 1816(27.56%) | 1199(25.40%) | 1062(24.51%) | 1175(24.26%) | <0.001 |
| High school | 1452(22.03%) | 1043(22.09%) | 993(22.92%) | 1182(24.40%) |  |
| More than high school diploma | 3322(50.41%) | 2479(52.51%) | 2278(52.57%) | 2487(51.34%) |  |
| Marital status |  |  |  |  |  |
| Married or living with partner | 4110(62.37%) | 2783(58.95%) | 2541(58.64%) | 2768(57.14%) | <0.001 |
| Widowed or divorced or separated | 1488(22.58%) | 1017(21.54%) | 949(21.90%) | 1133(23.39%) |  |
| Never married | 992(15.05%) | 921(19.51%) | 843(19.46%) | 943(19.47%) |  |
| BMI (kg/m^2^) |  |  |  |  |  |
| Under/Normal weight (≤24.9) | 1604(24.34%) | 1385(29.34%) | 1373(31.69%) | 1662(34.31%) | <0.001 |
| Overweight (25-29.9) | 2180(33.08%) | 1547(32.77%) | 1521(35.10%) | 1633(33.71%) |  |
| Obese (≥30) | 2806(42.58%) | 1789(37.89%) | 1439(33.21%) | 1549(31.98%) |  |
| Smoking |  |  |  |  |  |
| Yes | 2916(44.25%) | 2133(45.18%) | 1982(45.74%) | 2246(46.37%) | 0.137 |
| No | 3674(55.75%) | 2588(54.82%) | 2351(54.26%) | 2598(53.63%) |  |
| Diabetes |  |  |  |  |  |
| Yes | 801(12.15%) | 516(10.93%) | 532(12.28%) | 657(13.56%) | 0.001 |
| No | 5789(87.85%) | 4205(89.07%) | 3801(87.72%) | 4187(86.44%) |  |
| Hypertension |  |  |  |  |  |
| Yes | 2297(34.86%) | 1618(34.27%) | 1544(35.63%) | 1907(39.37%) | <0.001 |
| No | 4293(65.14%) | 3103(65.73%) | 2789(64.37%) | 2937(60.63%) |  |
| Fat (g) | 77.90±44.00 | 79.55±45.83 | 79.15±48.92 | 78.46±47.45 | 0.271 |
| SFA (g) | 24.96±15.47 | 25.49±16.08 | 25.69±17.70 | 25.55±17.32 | 0.660 |
| MUFA (g) | 28.12±16.79 | 28.73±17.66 | 28.48±18.77 | 28.19±17.92 | 0.184 |
| PUFA (g) | 17.85±11.88 | 18.22±12.24 | 17.92±12.37 | 17.75±12.21 | 0.256 |
| VB6 (mg) | 1.95±1.35 | 2.09±1.71 | 2.08±1.61 | 2.12±1.77 | <0.001 |
| VB12 (µg) | 4.99±7.48 | 5.02±5.52 | 5.09±5.99 | 5.24±6.15 | 0.207 |
| VC (mg) | 83.20±95.43 | 85.00±97.53 | 88.33±107.65 | 85.38±94.46 | 0.106 |
| VD(µg) | 4.41±5.23 | 4.56±5.26 | 4.8±6.16 | 4.81±5.87 | <0.001 |
| Folate(µg) | 390.08±230.26 | 404.70±257.92 | 397.05±243.34 | 402.28±262.50 | 0.308 |
| Caffeine (mg) | 148.63±198.74 | 156.10±200.26 | 156.45±216.68 | 155.95±216.47 | 0.228 |
| HDL (mmol/L) | 1.32±0.39 | 1.35±0.40 | 1.37±0.42 | 1.40±0.44 | <0.001 |
| HBV infection status |  |  |  |  |  |
| Positive | 61(0.93%) | 43(0.91%) | 31(0.72%) | 28(0.58%) | 0.141 |
| Negative | 6529(99.07%) | 4678(99.09%) | 4302(99.28%) | 4816(99.42%) |  |

Serum calcium was grouped by quartile.

Abbreviation: BMI, body mass index; HBV, Hepatitis B Virus; SFA, Saturated fatty acids; MUFA, Monounsaturated fatty acids; PUFA, Polyunsaturated fatty acids; VB6, vitamin B6; VB12, vitamin B12; VC, vitamin C; VD, vitamin D; HDL, high-density lipoprotein.
